# Supplementary material for: Quick, Coordinated and Authentic Reprogramming of Ribosome Biogenesis during iPSC Reprogramming
Source: Cells. 2020 Nov 15;9(11):2484. doi: 10.3390/cells9112484 (PMC7697288; doi:10.3390/cells9112484)
Supplement: Supplementary file 1 [file cells-09-02484-s001.zip › supplementary/cells-09-02484-s001.pdf]

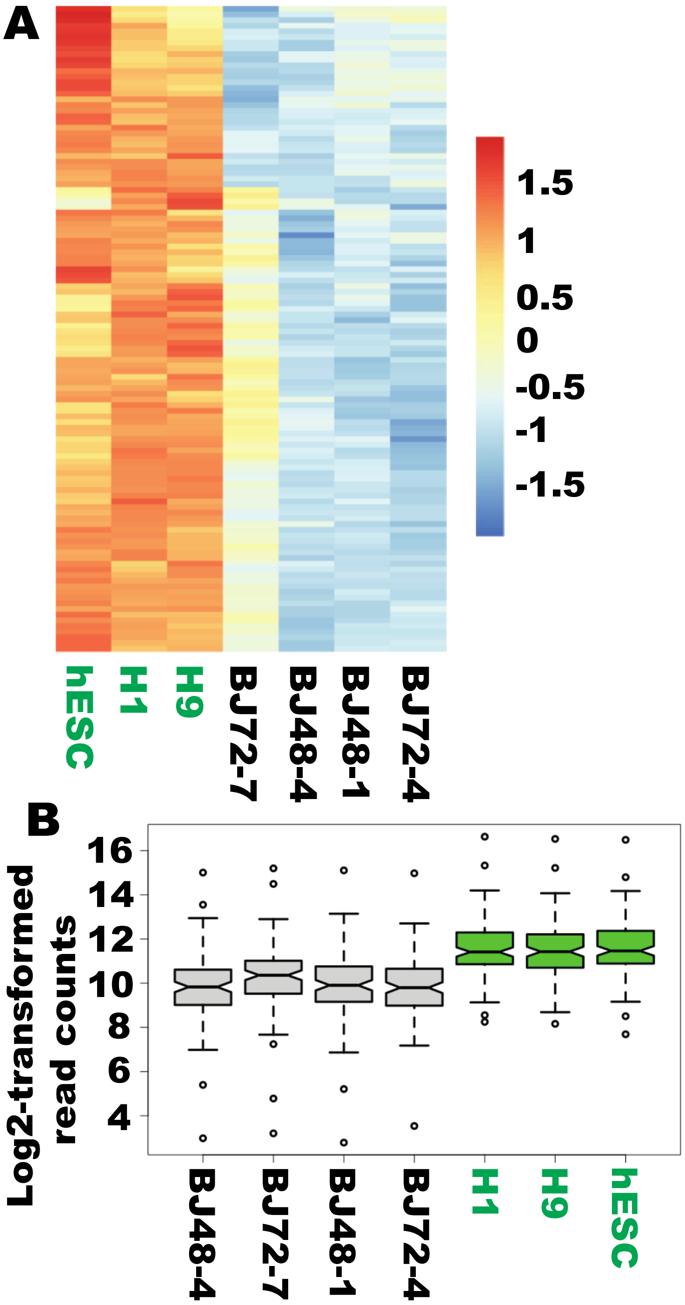

Figure S1. (Related to Figure 1). Out of the 291 human RB genes, 11 were enriched at least twofold in hESCs compared to fibroblasts. (A) A heat map showing enrichment of the 114 ribosome genes in hESCs. (B) Box plots for data in (A), but the sample average expression levels are shown. RNA-seq samples, hESC = 3 (green); fibroblasts = 4 (black). H1 and H9, human embryonic stem cell (ESC) line H1 and H9, respectively; hESC is a subline of H1. BJ, human primary fibroblast BJ cells. The first number after BJ indicates the harvest time post-seeding (48 and 72 h), and the second number is lane number of the flow cells for RNA-seq.

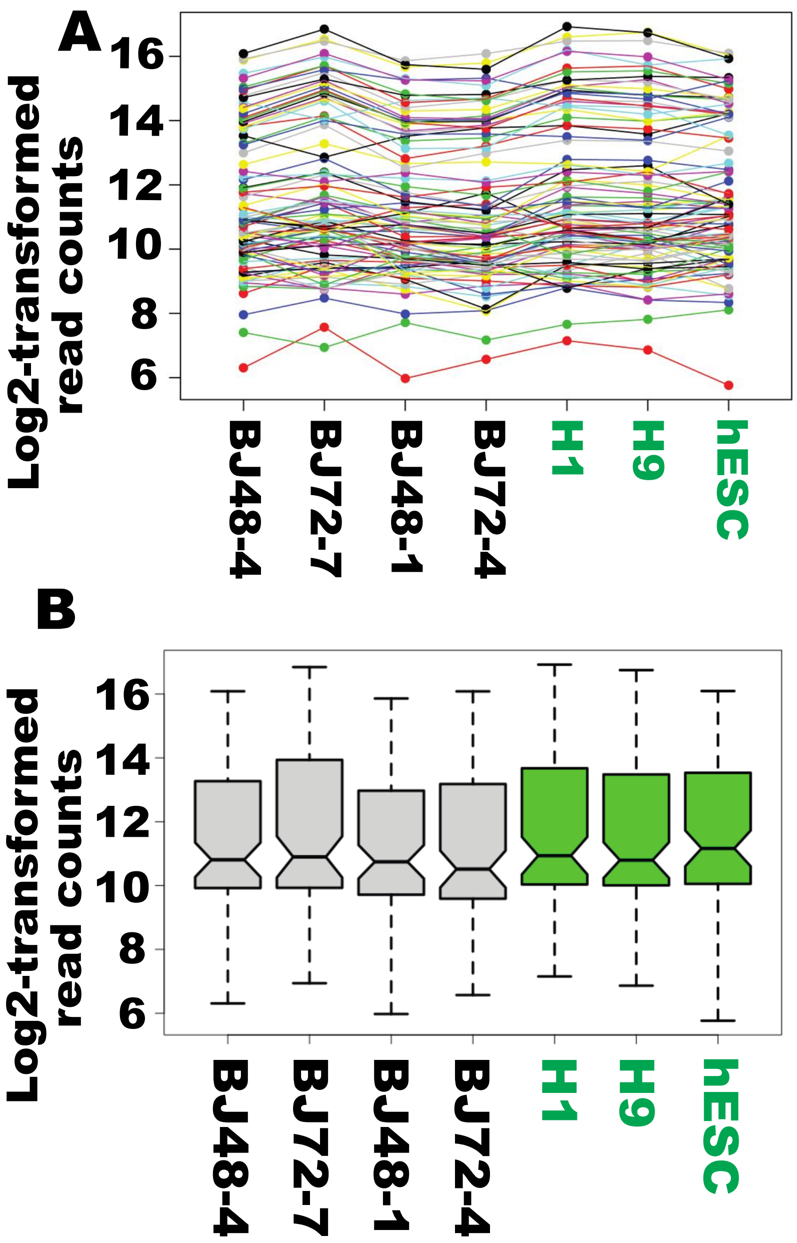

Figure S2. (Related to Figure S2. Out of the 291 human RB genes, 96 were expressed at similar levels in both cell types. (A) Ladder plots showing similar expression of the 96 human RB genes in both cell types. (B) Box plots for data in (A). The boxes and labels of hESCs are highlighted in green. Sample labels are the same as in Figure S1.

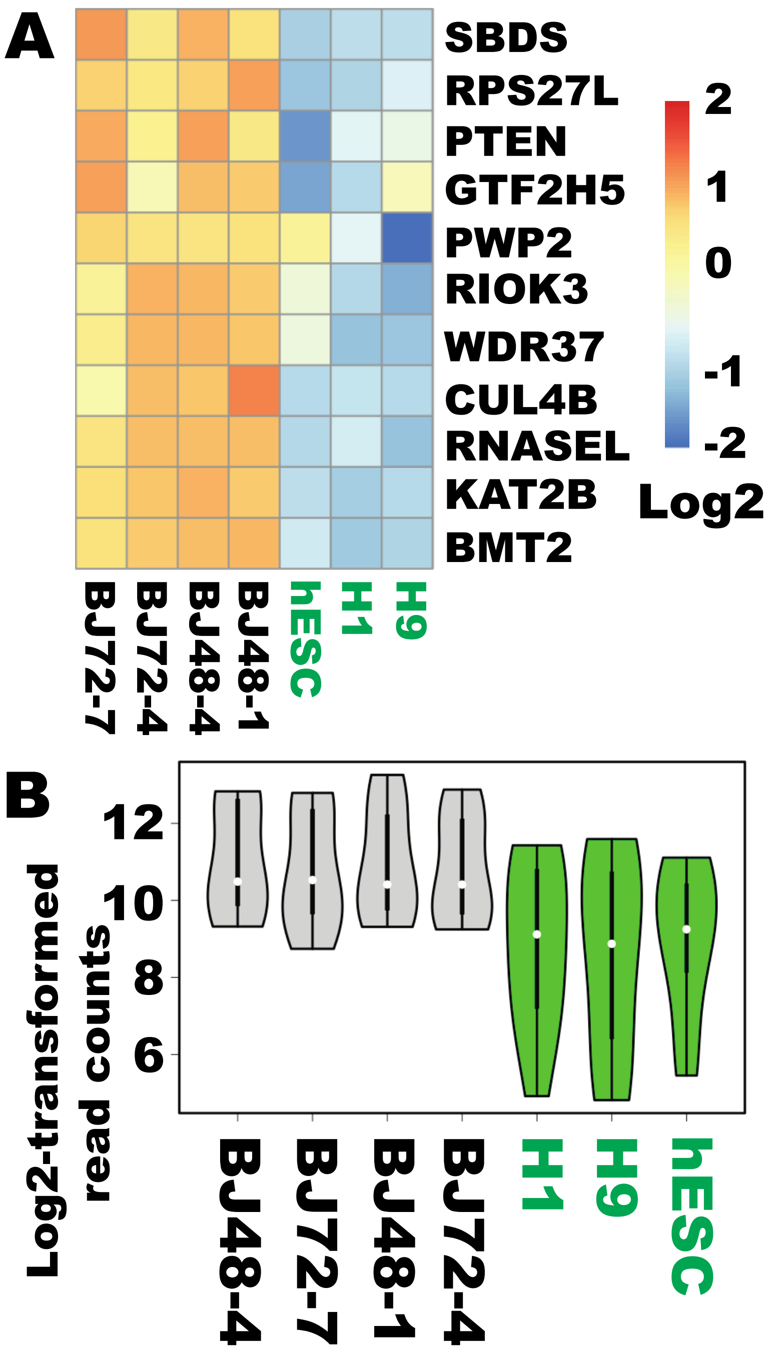

Figure S3. (Related to Figure 1). Out of the 291 human RB genes, 11 were expressed at least 2× higher in fibroblasts than in hESCs. (A) A heat map showing enrichment of the 11 RB genes in human fibroblasts. (B) Violin plots for data in (A). Sample labels are the same as in Figure S1.

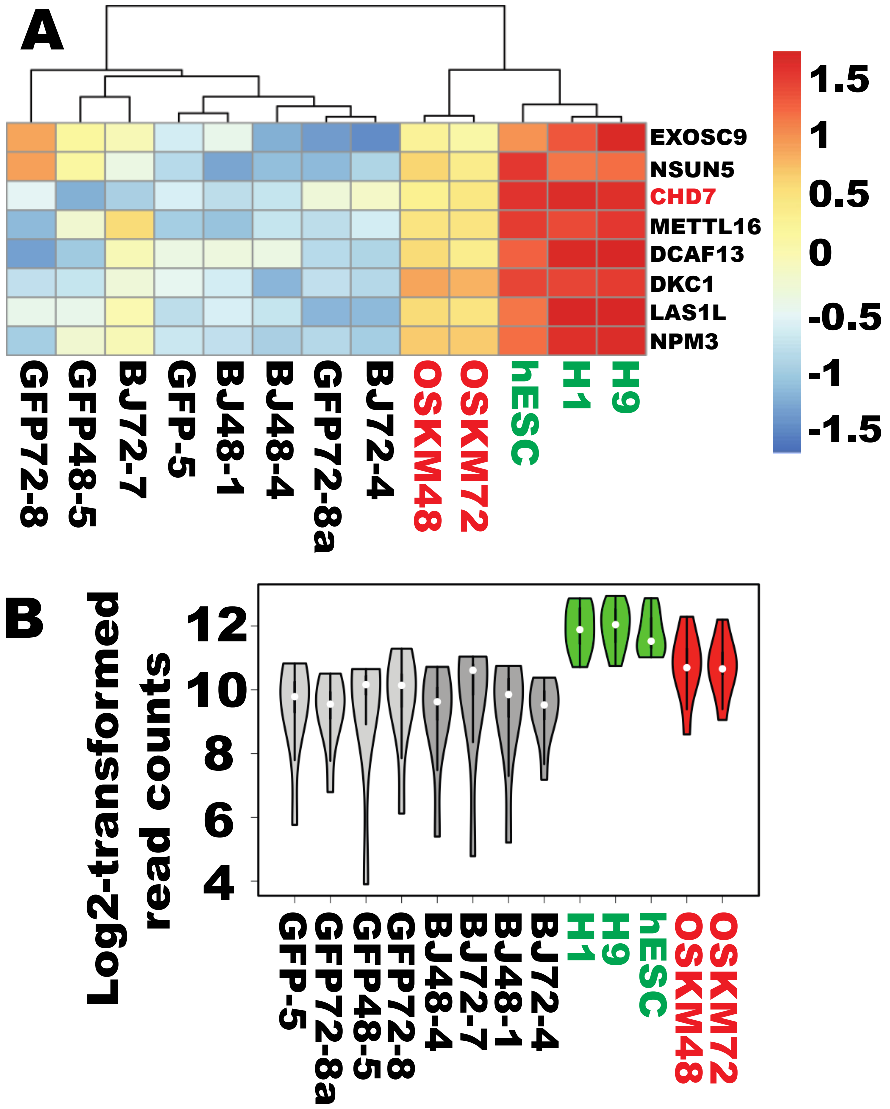

Figure S4. (Related to Figure 2). The eight hESC-enriched RB genes were insufficiently reprogrammed but significantly upregulated so that they became more similar to hESCs and dissimilar to the starting fibroblasts. (A) A heat map and clustering showing significant upreprogramming, albeit insufficiently, of the eight hESC-enriched RB genes. (B) Violin plots for the data in (A). OSKM: OCT4, SOX2, KLF4 and MYC; 48 and 72 after OSKM indicate hours post-OSKM transduction. Fibroblast samples transduced with OSKM are highlighted in red. Other sample labels

are the same as in Supplementary Figure 1.

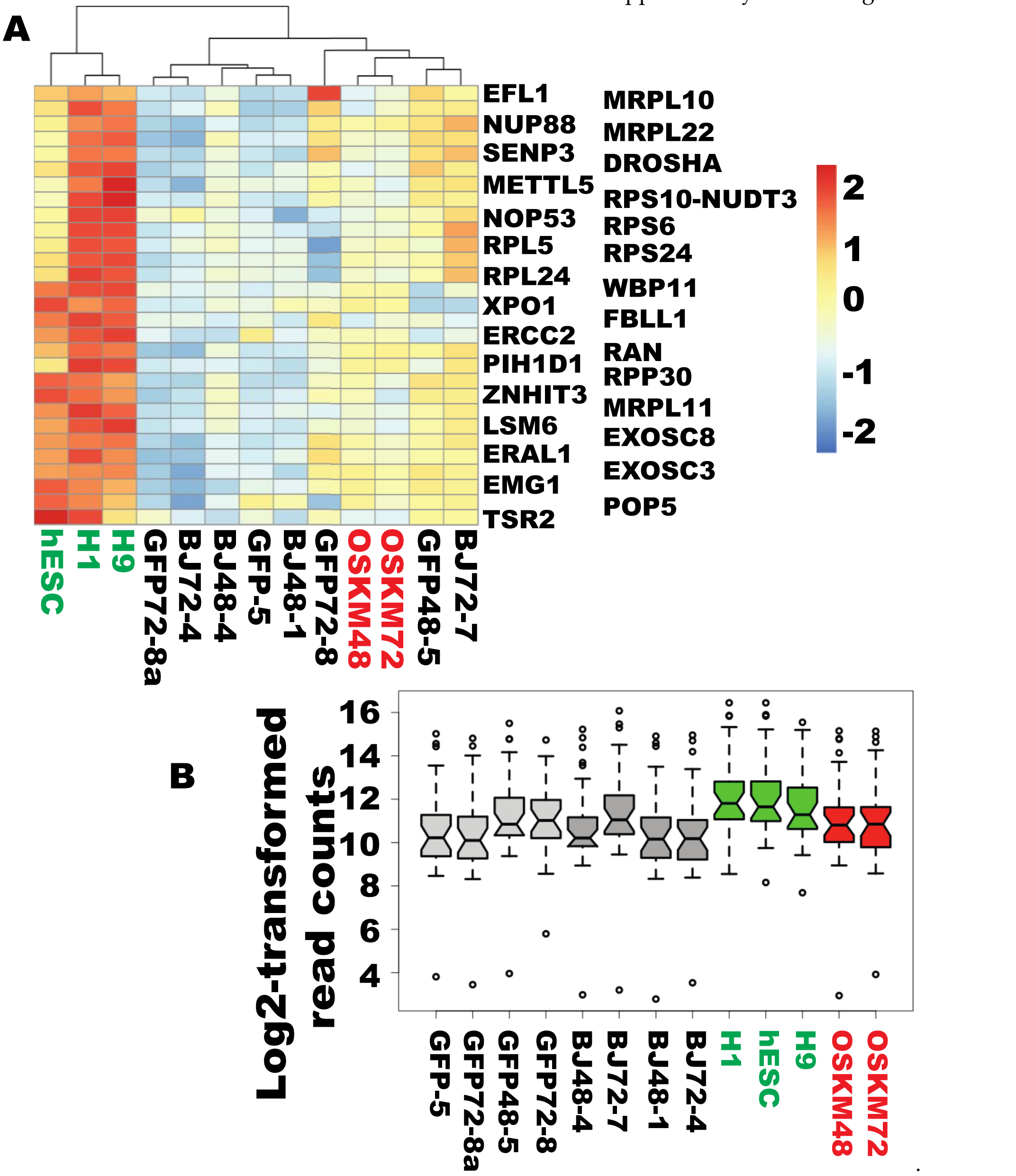

Figure S5. (Related to Figure 2). A total of 29 hESC-enriched RB genes were resistant to OSKM reprogramming at the early stages. (A) A heat map showing that 29 hESC-enriched RB genes were irresponsive to OSKM reprogramming. (B) Box plots for data in (A). Sample labels are the same as in Supplementary Figure 4.

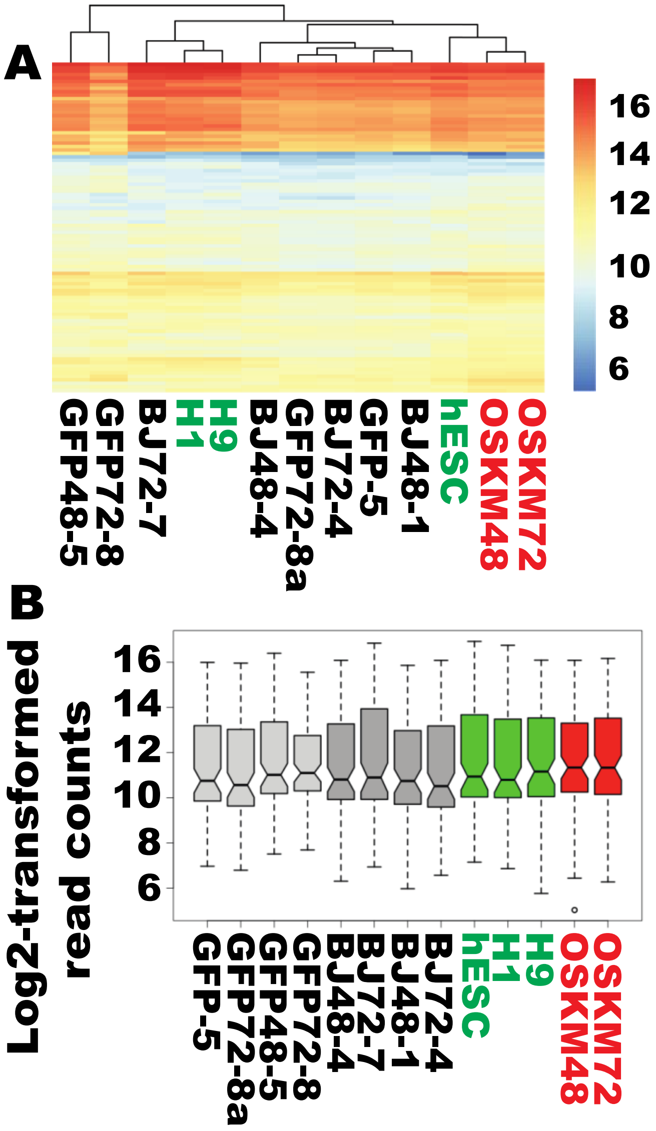

Figure S6 (Related to Figure 2). No aberrant reprogramming for the 96 shared RB genes between hPSCs and the starting fibroblasts. (A) A heat map showing similar expressions of the 96 shared RB genes across cell types and treatments, and random clustering of cell types and treatments. (B) Box plots showing similar expressions of the 96 RB genes across all samples. Sample labels are the same as in Supplementary Figure 4.

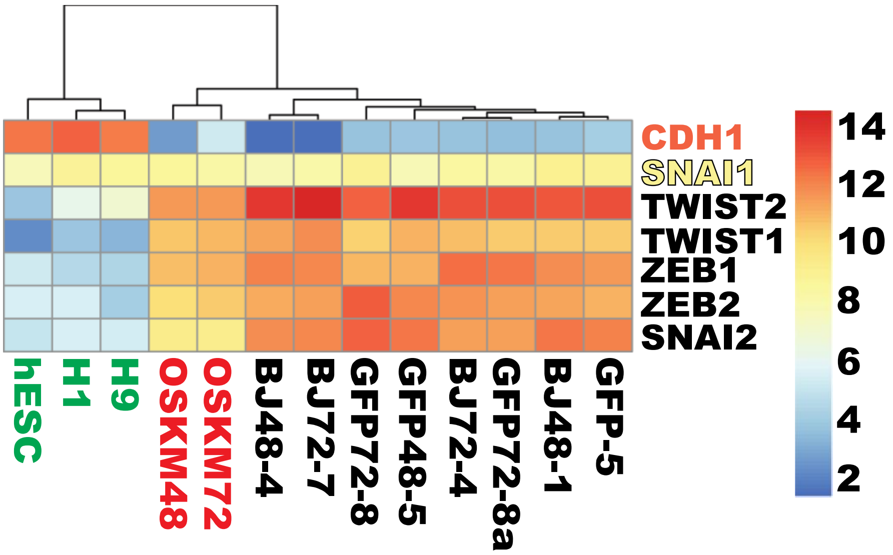

Figure S7. (Related to Figure 3). Key fibroblast-specific mesenchymal TFs and the hESC-specific epithelial marker gene *CDH1* were not reprogrammed at the time of proper RB reprogramming. The heat map shows little transcriptional responses of the seven MET key factors. The shared TF gene *SNAI1* and the gene coding for the epithelial marker e-cadherin *CDH1* are highlighted in the heat map. Sample labels are the same as in Supplementary Figure 4.

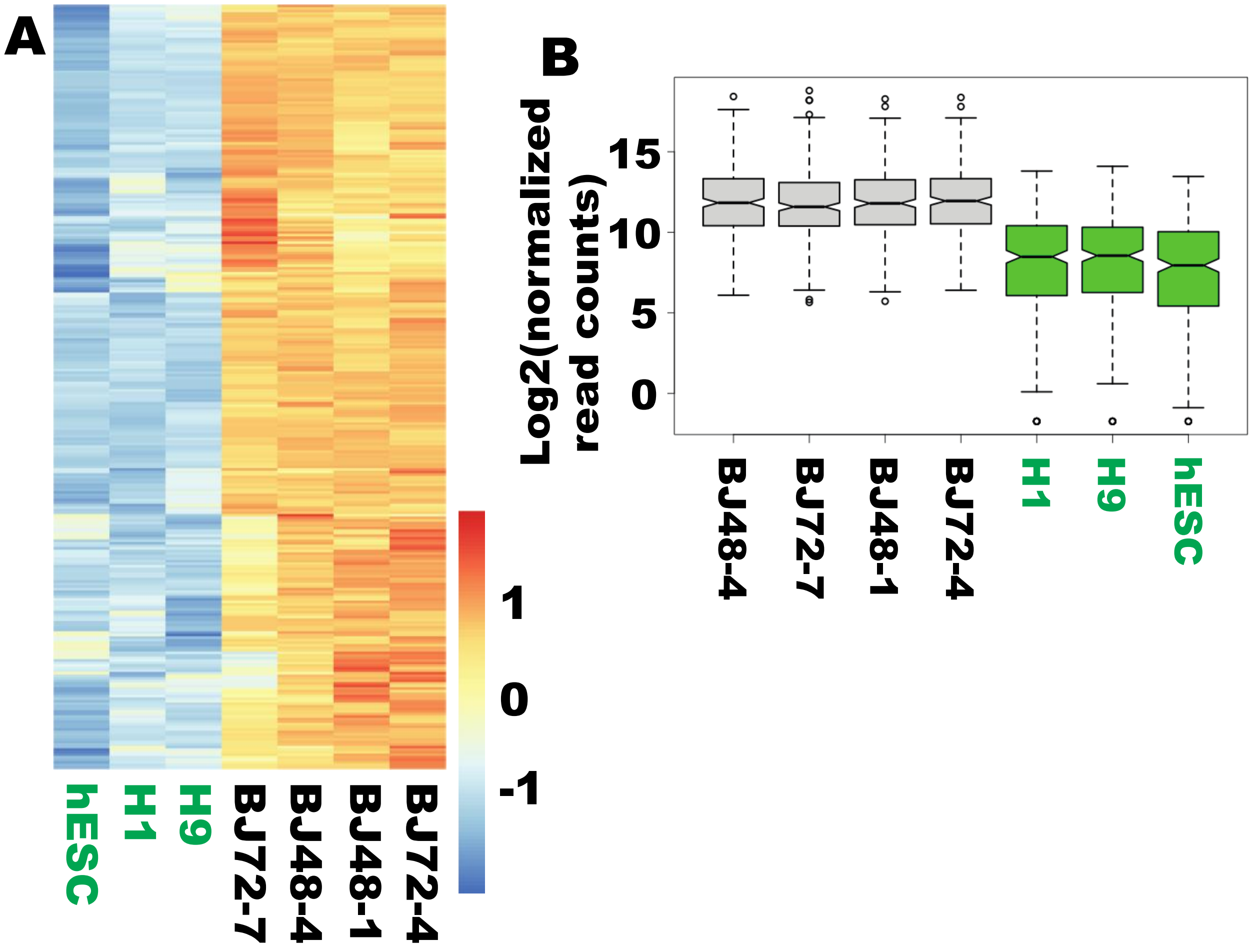

Figure S8. (Related to Figure 4). Downreprogrammome of 300 MET/EMT genes that were enriched in human fibroblasts compared to hPSCs. (A) Heat map showing higher expression of the 300 MET/EMT genes in human fibroblasts on the basis of log2-transformed read counts and scaled across samples for each gene. (B) Box plots for data in (A) but on the basis of the averaged normalized read counts to show overall higher expression of the 300 MET/EMT genes as a group in fibroblasts. Fibroblast = 4; hESC = 3.

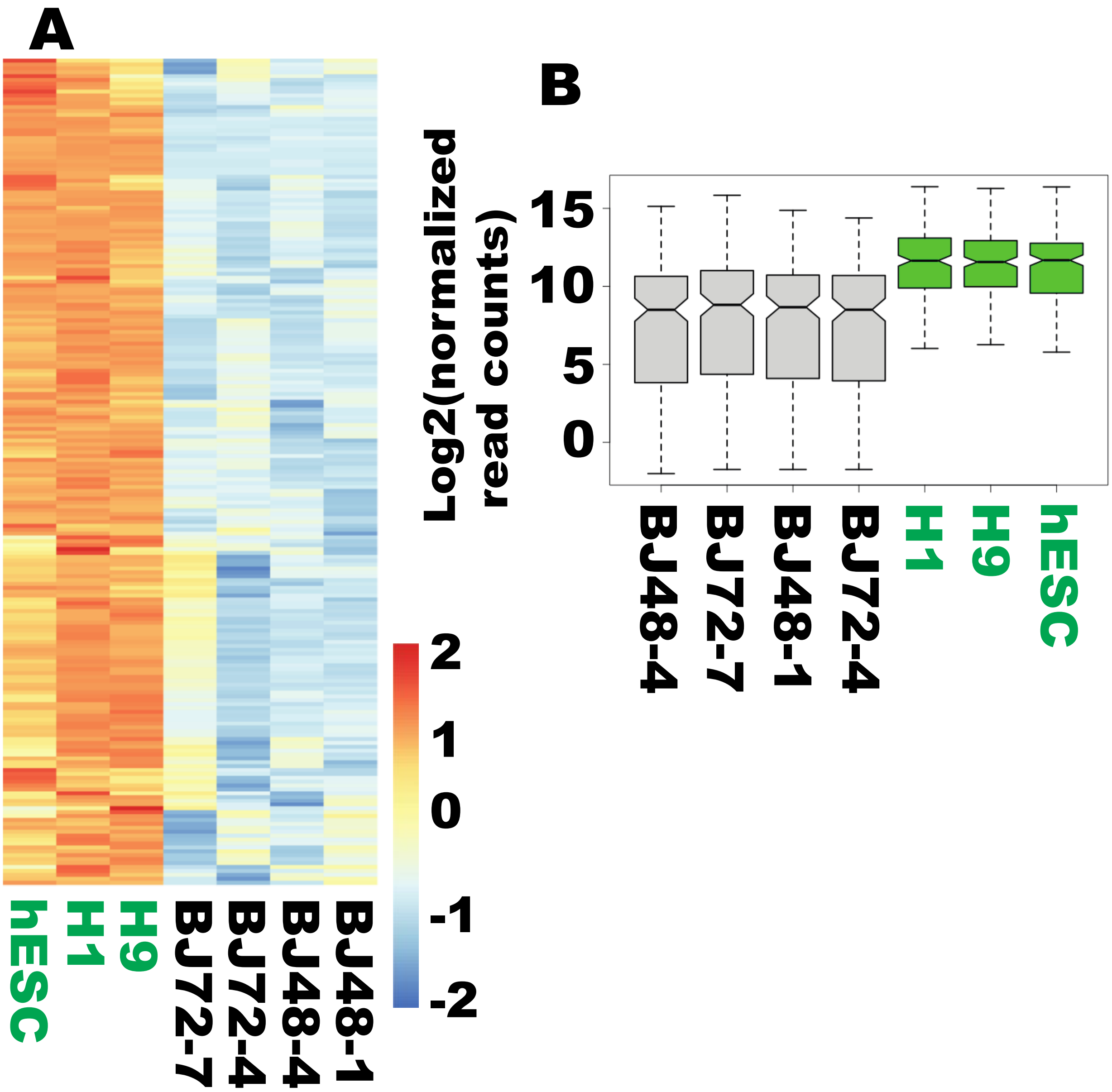

Figure S9. (Related to Figure 4). The upreprogramome of 213 MET/EMT genes that were enriched in human embryonic stem cells when compared to fibroblasts. (A) A heat map showing higher expression in hESCs for the 213 MET/EMT genes than in fibroblasts. (B) Box plots for data in (A) but on the basis of the averaged normalized read counts to show overall higher expression of the 213 MET/EMT genes as a group. Fibroblast = 4; hESC = 3.

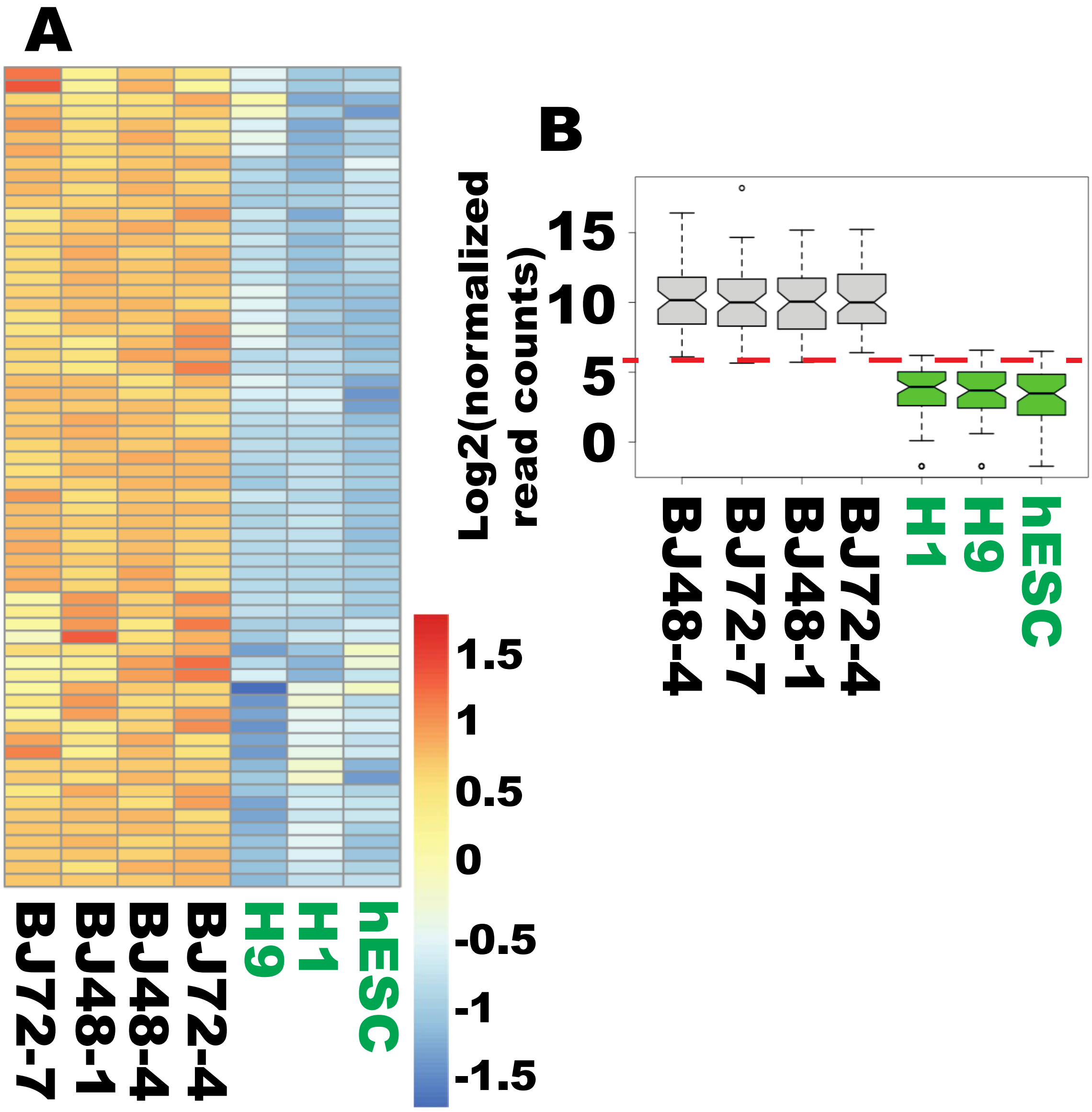

Figure S10. (Related to Figure 4). The **erasome of 64 MET/EMT genes that were expressed in human fibroblasts only, but not in hESCs**. (A) A heat map showing higher expressions for the 64 MET/EMT genes in fibroblasts than in hESCs. (B) Box plots for data in (A) but on the basis of the averaged normalized read counts to show high expression of the 64 MET/EMT genes as a group in fibroblasts, as well as lack of their expression in hESCs. Fibroblast = 4; hESC = 3. The dashed red line in (B) indicates the level of expression threshold for the expressed genes.

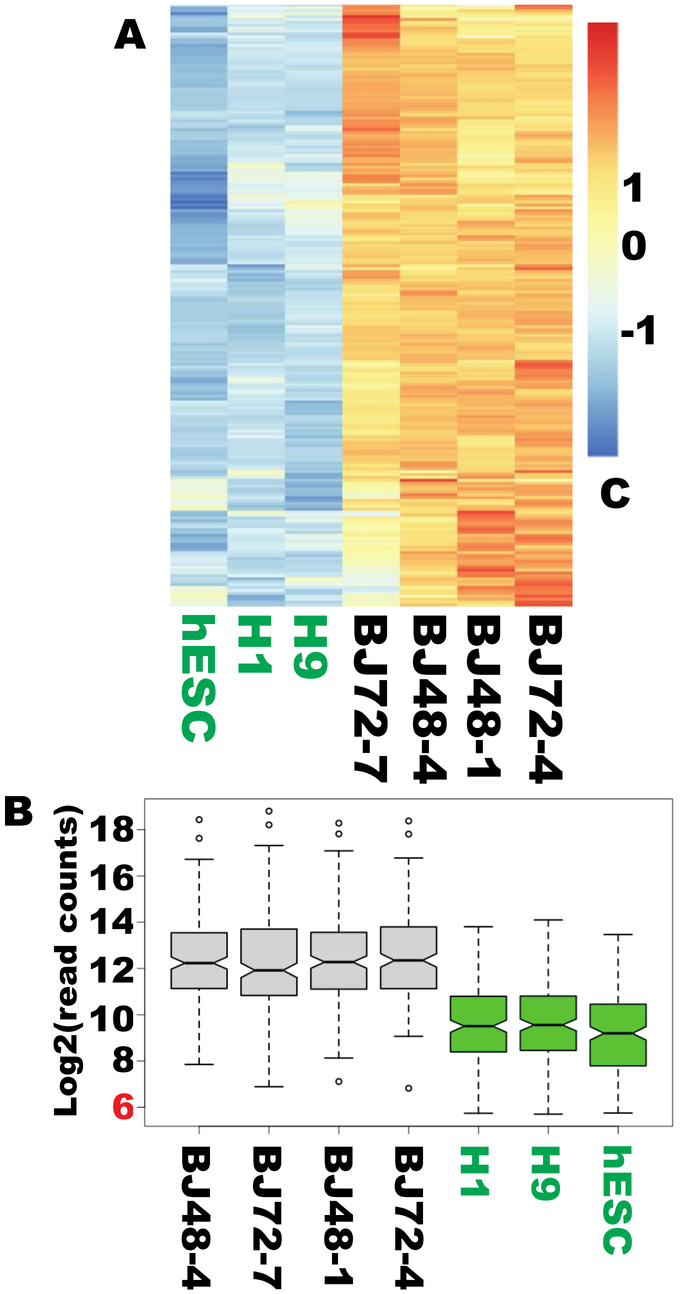

Figure S11. (Related to Figure 4). A total of 208 MET/EMT genes were expressed in both cell types, but were at least 2× higher in fibroblasts than in in hESCs. (A) A heat map showing higher expressions for the 208 MET/EMT genes in fibroblasts than in hESCs. (B) Box plot for data in (A) but on the basis of the averaged normalized read counts to show higher expressions of the 208 MET/EMT genes as a group in fibroblasts in comparison with those of hESCs. Fibroblast = 4; hESC = 3; FC > 2; q < 0.01. Note that the lowest log2 read counts on the *y*-axis is highlighted in red in (B), which is greater than the threshold value for the expressed genes.

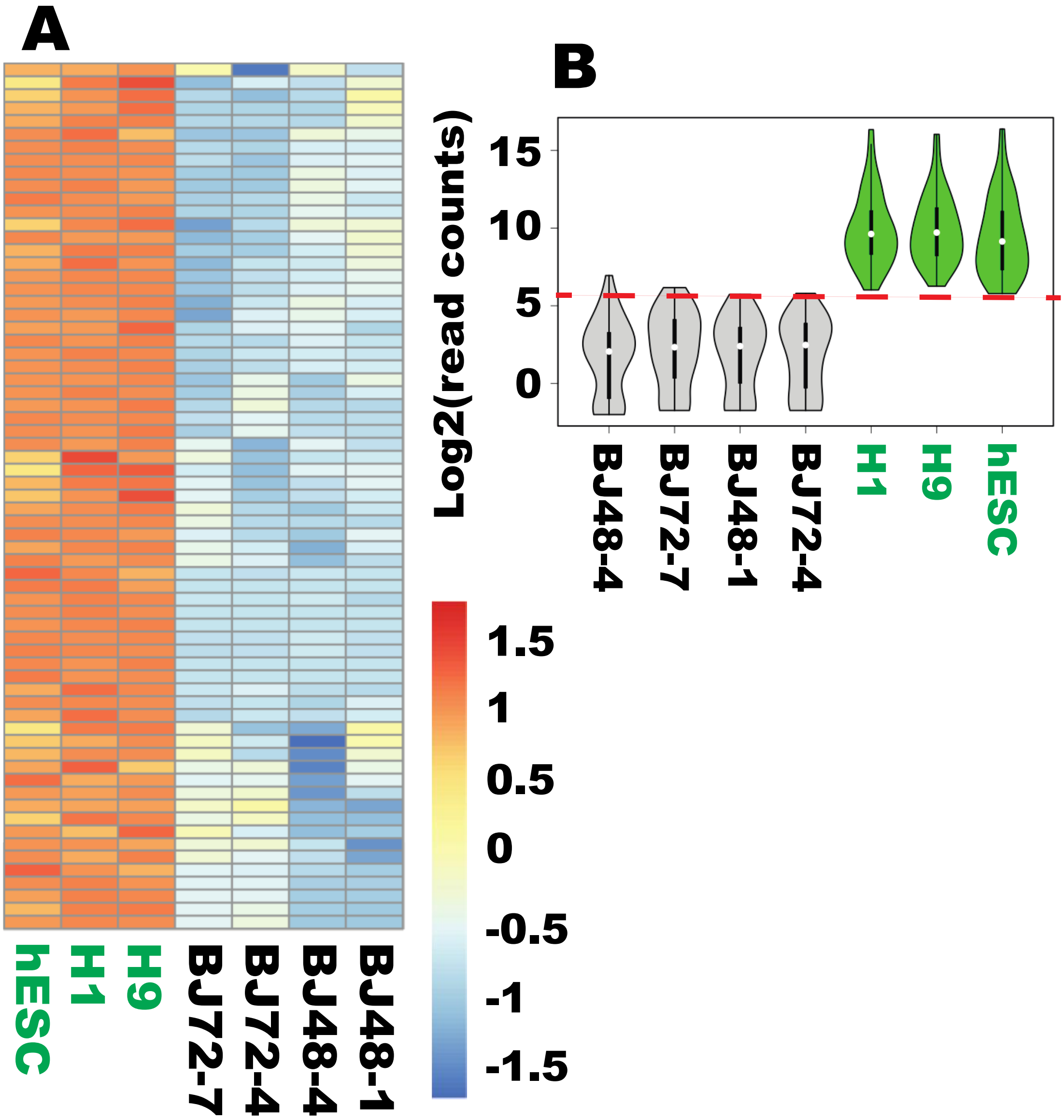

Figure S12. (Related to Figure 4). Activatome of 67 MET/EMT genes that were expressed in hESCs only, but not in fibroblasts. (A) A heat map showing higher expression for the 67 MET/EMT genes in hESCs than in fibroblasts. (B) Violin plots for data in (A) but based on the averaged normalized read counts to show expression of the 67 MET/EMT genes as a group in hESCs, but lack of expression in fibroblasts. Fibroblast = 4; hESC = 3. The red broken line in (B) indicates the level of the expression threshold.

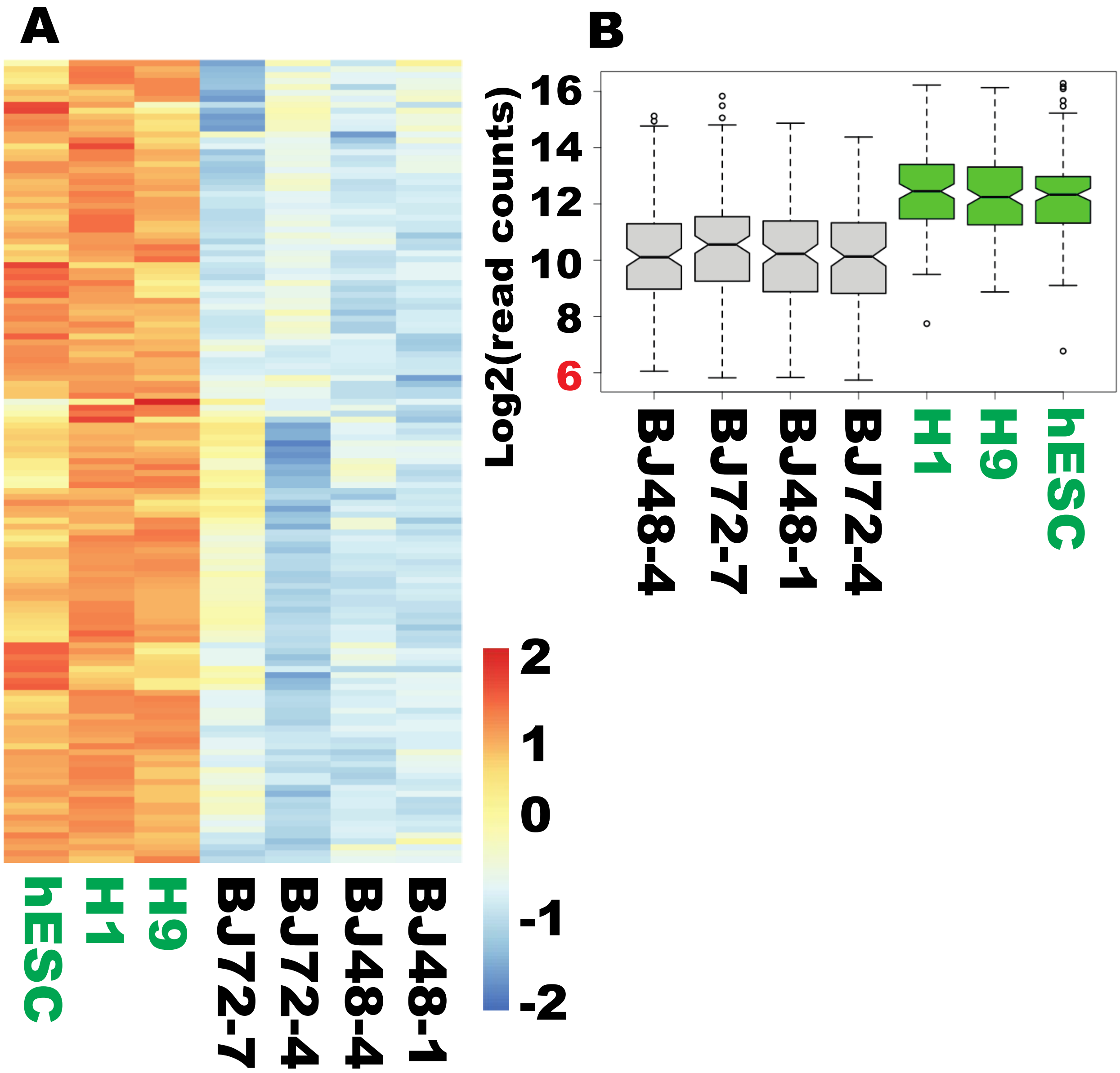

Figure S13. (Related to Figure 4). A total of 135 MET/EMT genes were expressed in both cell types, but at least 2× higher in in hESCs than in fibroblasts. (A) Heat map showing higher expression for the 135 MET/EMT genes in hESCs than in fibroblasts. (B) Box plots for data in (A) but on the basis of the averaged normalized read counts to show higher expressions of the 135 MET/EMT genes as a group in hESCs in comparison with that of fibroblasts. Fibroblast = 4; hESC = 3; FC > 2; q < 0.01. Note that the lowest log2 scale on the *y*-axis is highlighted in red in (B), which is greater than the threshold value for the expressed genes.

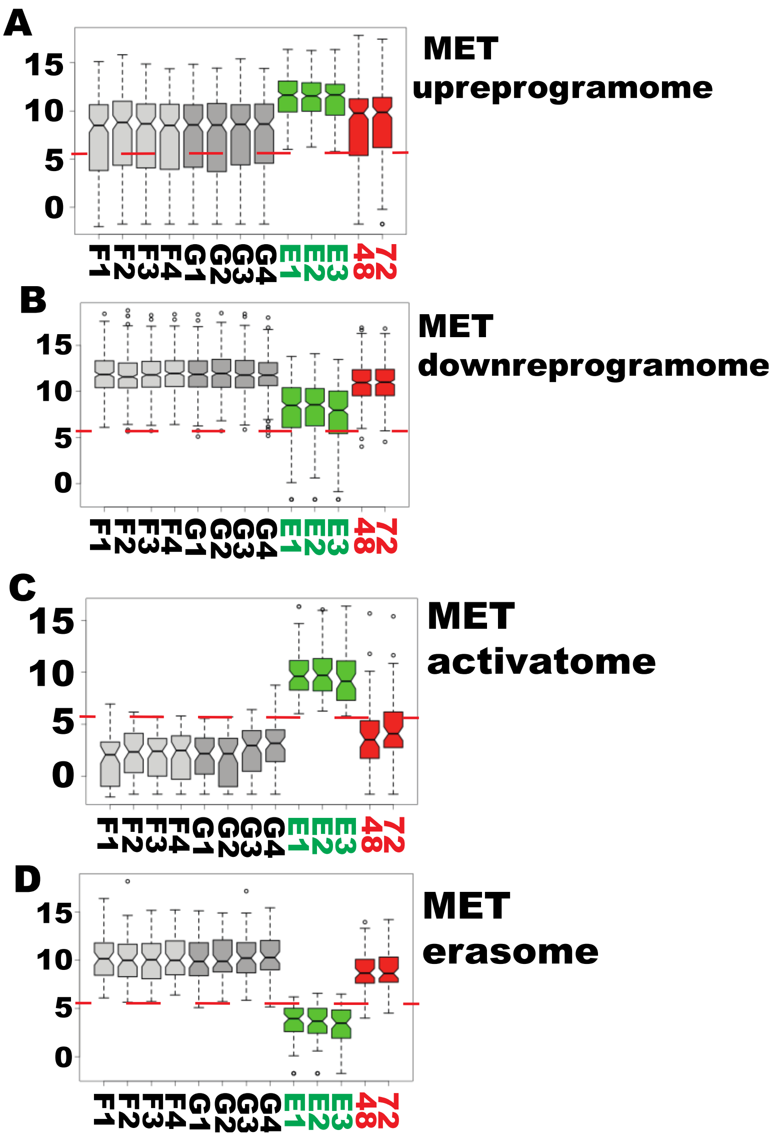

Figure S14. None of the MET subreprogramomes were reprogrammed at the time of proper reprogramming of ribosome biogenesis. MET upreprogramome, downreprogramome, activatome, and erasome were not reprogrammed, as visualized by box plots (A)–(D). Dashed red lines indicate the threshold value of the expressed genes.

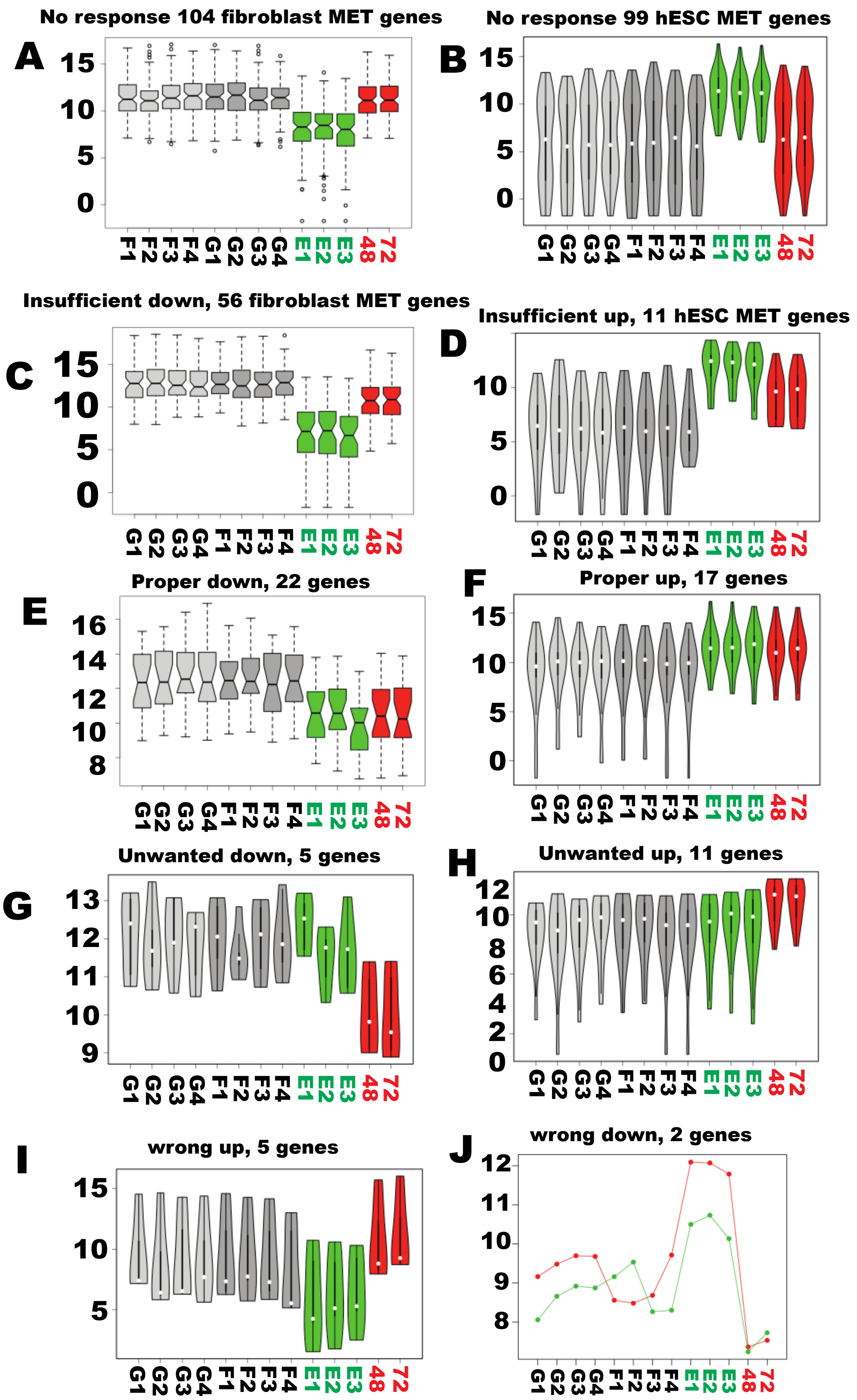

Figure S15. (Related to Figure 6). A PIANO response of the MET/EMT genes to the reprogramming factors OSKM at the initial stages of pluripotency reprogramming. Each category was visualized as box plots, violin plots, or ladder plots. (A) A total of 104 fibroblast-enriched MET genes did not respond to OSKM induction at both time points (48 h and 72 h); (B) 99 hESC-enriched genes did not

respond to OSKM reprogramming at the initial stages; (C) 56 fibroblast-enriched MET genes were significantly downregulated by at least twofold, but insufficiently downreprogrammed with a deficiency of at least twofold; (D) 11 hESC-enriched MET genes were significantly upregulated by at least twofold, but insufficiently upreprogrammed with a deficiency of at least twofold; (E) 22 fibroblast-enriched MET genes were properly downreprogrammed to the levels of hESCs within 48 h post-OSKM transduction; (F) 17 hESC-enriched genes were properly upreprogrammed to the levels of hESCs within 48 h; (G) 5 common MET genes were significantly downregulated by at least twofold when they should not have been; (H) 11 common MET genes were upregulated by at least twofold when they should not have been; (I) 5 fibroblast-enriched genes were wrongly upregulated by OSKM by at least twofold when they should have been downreprogrammed by at least twofold; (J) 2 hESC-enriched MET genes were downregulated significantly by at least twofold when they should have been upreprogrammed by at least twofold. G1 to G4: GFP-5, GFP72-8a, GFP48-5, and GFP72-8; F1 to F4: BJ48-4, BJ72-7, BJ48-1, and BJ72-4; E1 to E3: human embryonic stem cell H1, H9, and hESC; 48 and 72: RNA harvested from human fibroblasts 48 and 72 h post-transduction with lentiviral reprogramming factors OSKM.

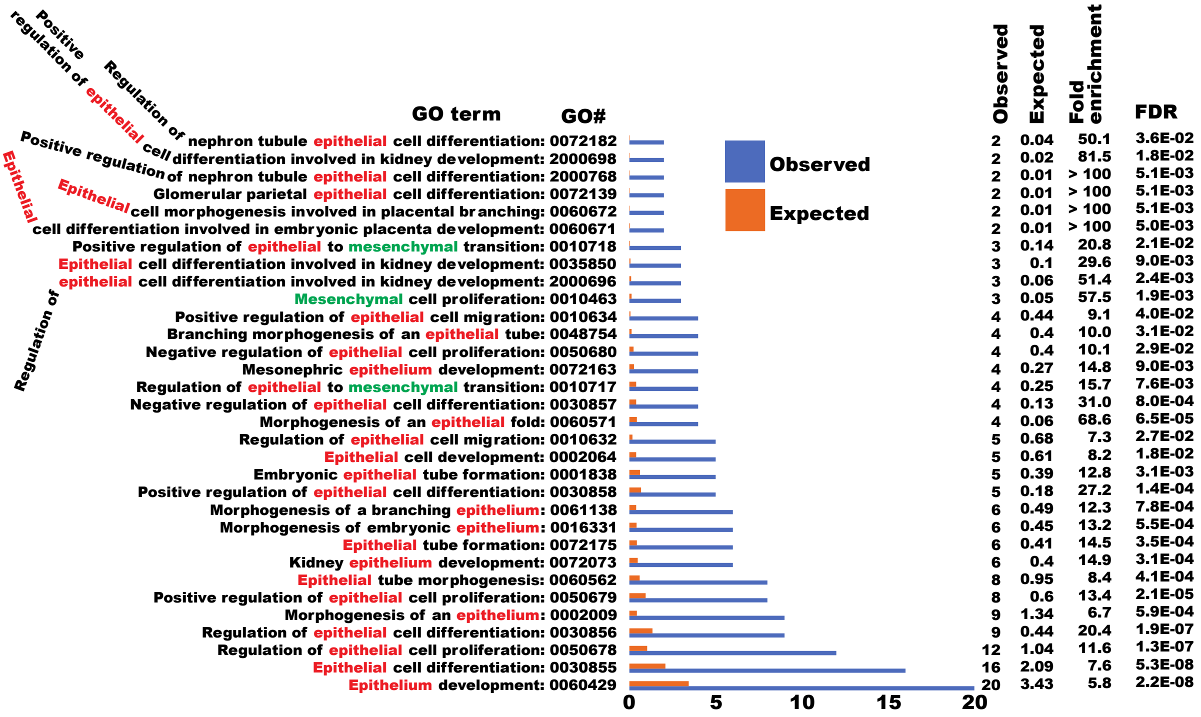

Figure S16. (Related to Figure 7). Summary of Gene Ontology (GO) terms with the key words of "epithelial" or "epithelium" overrepresented by the 67 hESC-specific MET/EMT genes. The key words of "epithelial" and "epithelium" are highlighted in red, while "mesenchymal" is highlighted in green.

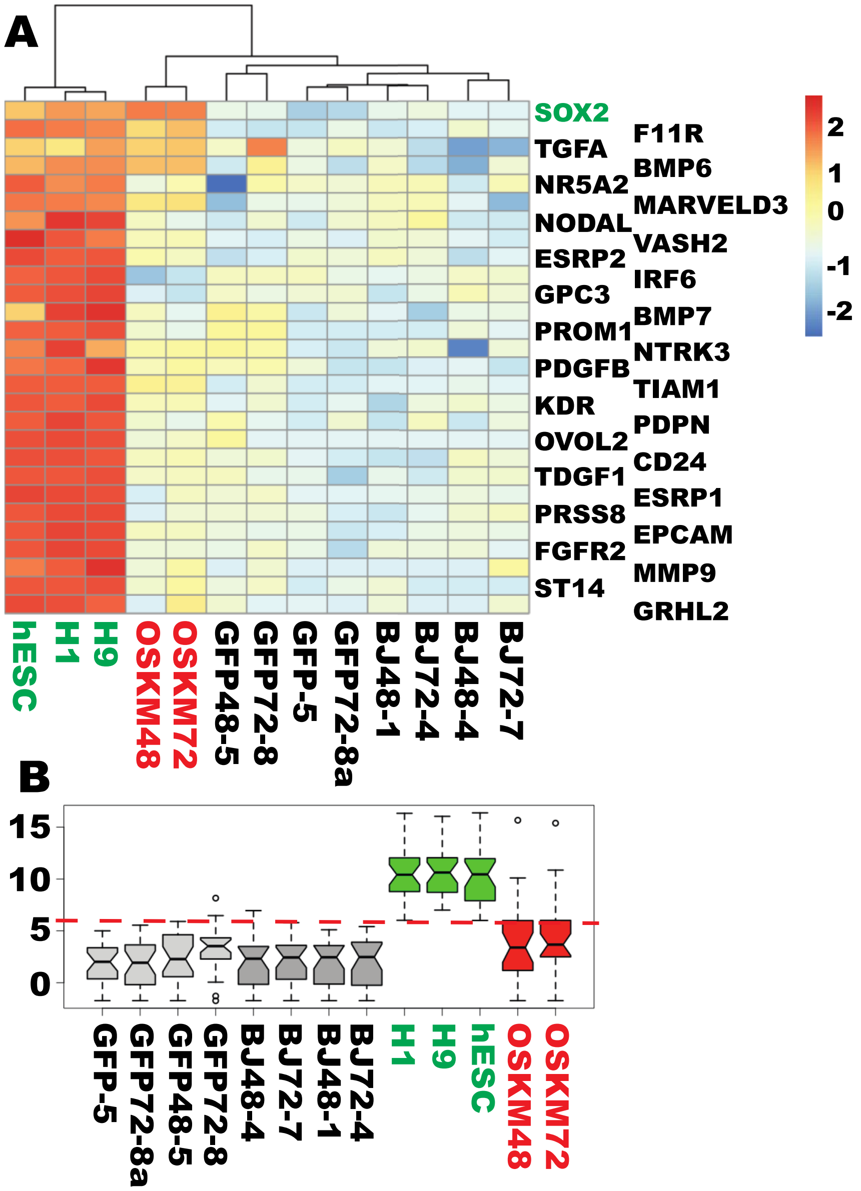

Figure S17. (Related to Figure 7). Of the 28 hESC-specific PANTHER-annotated epithelial genes, 25 were not reprogrammed at the time of proper reprogramming of ribosome biogenesis. (A) A heat map showing failure of reprogramming for the PANTHER-annotated epithelial genes at the initial stages of Yamanaka reprogramming. Note the clustering of reprogramming cells with the starting fibroblasts and the GFP-transduced control fibroblasts. (B) Box plots showing lack of reprogramming for the hESC-specific annotated epithelial genes. The dashed red line in (B) marks the threshold value for the expressed genes. The reprogramming factor of SOX2 is highlighted in green.

Supplementary Table 1. (Related to Figure 1). Summary for RNA-seq data of human ESCs and fibroblasts, and statistics for all the 298 human genes involved in ribosome biogenesis. Human ESC = 3, fibroblasts = 4.

Supplementary Table 2. (Related to Figure 1). Summary of RNA-seq data and the associated statistical data for the 114 human genes of ribosome biogenesis that were expressed at least twofold higher in hESCs than in fibroblasts. ESC = 3, fibroblasts = 4. $q < 0.01$.

Supplementary Table 3. (Related to Figure 1). Summary of RNA-seq and the associated statistical data for the 96 human genes of ribosome biogenesis that were expressed in both cell types at similar levels. ESC = 3, fibroblasts = 4. $FC < 1.5$.

Supplementary Table 4. (Related to Figure 1). Summary of RNA-seq data and the associated statistical data for the 11 human RB genes that were enriched in fibroblasts in comparison with that in hESCs. ESC = 3, fibroblasts = 4. $FC < 1.5$.

Supplementary Table 5. (Related to Figure 1). Summary of RNA-seq data and the associated statistical data for the 170 human RB genes that were enriched in hESCs by at least 1.5-fold compared with that in fibroblasts. ESC = 3, fibroblasts = 4. FC > 1.5x, q < 0.05.

Supplementary Table 6. (Related to Figure 2). Normalized RNA-seq read counts and the related data showing that only 2 of the 11 fibroblast-enriched RB genes were properly reprogrammed at the early stage of OSKM induction. ESC = 3, fibroblasts = 4, GFP = 4; OSKM = 2.

Supplementary Table 7. (Related to Figure 2). The normalized RNA-seq read counts and the related data for the 170 hPSC-enriched RB genes at the early stage of OSKM reprogramming. ESC = 3, fibroblasts = 4, GFP = 4; OSKM = 2.

Supplementary Table 8. (Related to Figure 2). Normalized RNA-seq read counts, the related metadata, and statistic data for the eight hPSC-enriched RB genes that were insufficiently upreprogrammed, but significant upregulations were achieved at the early stage of OSKM reprogramming. ESC = 3, fibroblasts = 4, GFP = 4; OSKM = 2.

Supplementary Table 9. (Related to Figure 2). Normalized RNA-seq read counts, and the related metadata and statistic data for the 29 hPSC-enriched RB genes that did not respond to OSKM induction at the early stage of OSKM reprogramming. ESC = 3, fibroblasts = 4, GFP = 4; OSKM = 2.

Supplementary Table 10. (Related to Figure 2). Normalized RNA-seq read counts and the related data for the 96 common ribosome biogenesis genes showing that their expressions were generally not impacted by OSKM reprogramming, i.e., no aberrant reprogramming. ESC = 3, fibroblasts = 4, GFP = 4; OSKM = 2.

Supplementary Table 11. (Related to Figure 3). Normalized RNA-seq read counts and the related data for the seven key genes involved in MET, showing that these genes were not reprogrammed at the time of proper RB reprogramming. ESC = 3, fibroblasts = 4, GFP = 4; OSKM = 2. The statistics data were for the comparison of OSKM/GFP.

Supplementary Table 12. Normalized RNA-seq read counts, metadata, and the related statistical data for the 300 MET/EMT genes enriched in human fibroblasts when compared to hPSCs. ESC = 3; fibroblasts = 4; q(FDR) < 0.01; FC >2.

Supplementary Table 13. Normalized RNA-seq read counts, metadata, and the related statistical data for the 213 MET/EMT genes enriched in hPSCs when compared to human fibroblasts. ESC = 3; fibroblasts = 4; q(FDR) < 0.01; FC >2.

Supplementary Table 14. Normalized RNA-seq read counts, metadata, and the related statistical data for the 613 MET/EMT with similar expression levels in both cell types. ESC = 3; fibroblasts = 4.

Supplementary Table 15. Normalized RNA-seq read counts, metadata, and the related statistical data for the 298 genes of ribosome biogenesis, showing that RB is globally enriched in human iPSCs (iPSC = 4, fibroblast = 4).

Supplementary Table 16. Normalized RNA-seq read counts, metadata, and the related statistical data for the 298 genes of ribosome biogenesis, showing that RB is globally enriched in human ESCs compared to an independent fibroblast line (fibroblast = 4, ESC = 4).

Supplementary Table 17. Normalized RNA-seq read counts, metadata, and the related statistical data for the 298 genes of ribosome biogenesis, showing that RB is globally reprogrammed in an independent fibroblast line (fibroblast = 4, OSKM = 4).
